# Bacteremia and adrenal gland abscess due to *Nocardia cyriacigeorgica*: a case report and review

**DOI:** 10.1186/s12879-022-07839-9

**Published:** 2022-12-29

**Authors:** Florian Saunier, Sylvain Grange, Josselin Rigaill, Marie-France Lutz, Amandine Gagneux-Brunon, Elisabeth Botelho-Nevers

**Affiliations:** 1 Infectious Disease Department, University Hospital of Saint Etienne, 42055 Saint-Étienne, France; 2 Department of Radiology, University Hospital of Saint Etienne, 42055 Saint-Étienne, France; 3 Laboratory of Infectious Agents and Hygiene, University Hospital of Saint-Etienne, 42055 Saint-Étienne, France

**Keywords:** *Nocardia cyriacigeorgica*, Nocardiosis, Adrenal gland abscess, Bacteremia

## Abstract

**Background:**

*Nocardia cyriacigeorgica* is one of the most common *Nocardia* species found in human infections, recently reclassified. Even though *Nocardia* may affect all organs by hematogenous dissemination, bacteremia are uncommon. Among all possible dissemination sites, the involvement of the adrenal glands is particularly rare.

**Case presentation:**

We report here a rare case of *Nocardia* disseminated infection with notably bacteremia and adrenal gland abscess, in a 77-years-old immunocompetent man. Adrenal gland abscess diagnosis was made by imaging (computerized tomography, magnetic resonance and positron emission tomography scan). A complete regression of all lesions including the left adrenal gland was obtained after 6 months of antibiotics. A review of literature was also performed.

**Conclusion:**

*Nocardia* bacteremia is a rare event but blood cultures may help to improve detection of *Nocardia* spp. in a non-invasive way. Adrenal abscess due to *Nocardia* spp. is very rare with only fourteen cases reported in the literature, but it is a true cause of adrenal masses. Our report suggests that clinician should be aware of this rare location and prioritize a non-invasive diagnosis strategy.

## Background

*Nocardia* is a genus of aerobic actinomycetes and belongs to the family of *Nocardiaceae* [1]. Bacteria are gram positive, branching, filamentous, and mildly acid-alcohol-fast [1, 2]. *Nocardia* species are ubiquitous, saprophytic and usual component of the soil, water and organic matter [1, 2]. Human infections arise mostly by inhalation and sometimes by skin inoculation [1, 2]. Currently, 123 *Nocardia* species are described according to the List of Prokaryotic names with Standing in Nomenclature (http://www.bacterio.net), and at least 50 are clinically significant [3]. Thanks to molecular methods (heat shock protein (hsp65) gene, 16 S rRNA gene sequencing), a significant taxonomic changes and species reassignment within the genus were made, particularly among members of the former *N. asteroides* complex [3]. *Nocardia asteroides* drug pattern type VI is now known as *Nocardia cyriacigeorgica* [3]. Thus, it might be difficult for clinicians to understand all the recent changes of *Nocardia* taxonomy.

Nocardiosis may be localized or disseminated and occur predominately in immunocompromised hosts [1]. The most common infection sites are the lungs, the central nervous system and the skin and soft tissues. All organs may potentially be affected by hematogenous dissemination but *Nocardia* spp. bacteremia are uncommon [1, 4, 5] and the involvement of the adrenal glands is particularly rare with only a few cases reported in the literature [6]. We report here a rare case of bacteremia and adrenal gland abscess due to *N. cyriacigeorgica*. This case is an opportunity to raise awareness of clinicians about the recent change of *Nocardia* taxonomy, and to review the frequency of bacteremic infection as well as adrenal gland abscesses during Nocardiosis.

## Case presentation

A 77-year-old man with no medical history presented for several months a deterioration of his general status with depressive syndrome. In November 2017, he was admitted to the emergency room for respiratory distress. Physical exam revealed the absence of fever, irregular cardiac rate and oxygen saturation at 95% with 9 L of oxygen. Electrocardiogram showed atrial fibrillation. Chest X-ray revealed interstitial syndrome of pulmonary bases. Laboratory investigations revealed leukocytes count 20.4 G/L with 18.60 G/L neutrophils, C-reactive protein 447 mg/L and serum creatinine level 314 µmol/L. He was treated as a severe pneumonia by intravenous levofloxacin (500 mg/12 h) and ceftriaxone (2 g/24 h). The blood pressure fell and the patient was transferred to an intensive care unit where he was intubated because of severe refractory hypoxemia despite high oxygen flow.

A chest CT-scan (computerized tomography) was performed and revealed a diffuse bilateral interstitial syndrome. Five set of blood cultures grew *Nocardia cyriacigeorgica* in aerobic bottles after respectively 67, 71, 81, 82 and 85 h (blood culture system BD BACTEC FX (Becton Dickinson®), identification was done using matrix-assisted laser desorption-ionization—time of flight mass spectrometry system (MALDI-TOF MS, Microflex LT, Bruker). Antibiotic susceptibility was determined using agar diffusion assay and ETEST® technique (bioMérieux), following the CLSI guidelines (https://clsi.org/). The isolate was susceptible to imipenem, cefotaxime, amikacin, clarithromycin, linezolid, doxycycline, and trimethoprim-sulfamethoxazole (TMP-SMX). It was non-susceptible to amoxicillin, ciprofloxacin and amoxicillin-clavulanic acid (intermediate). A diagnosis of disseminated Nocardiosis was subsequently made and antibiotic regimen was changed to linezolid (600 mg/12 h), cefotaxime (2 g/4 h) and amikacin (1.5 g/24 h). As the patient continued to work in the construction industry, we supposed that the source of Nocardiosis was probably by inhalation.

**Fig. 1:**
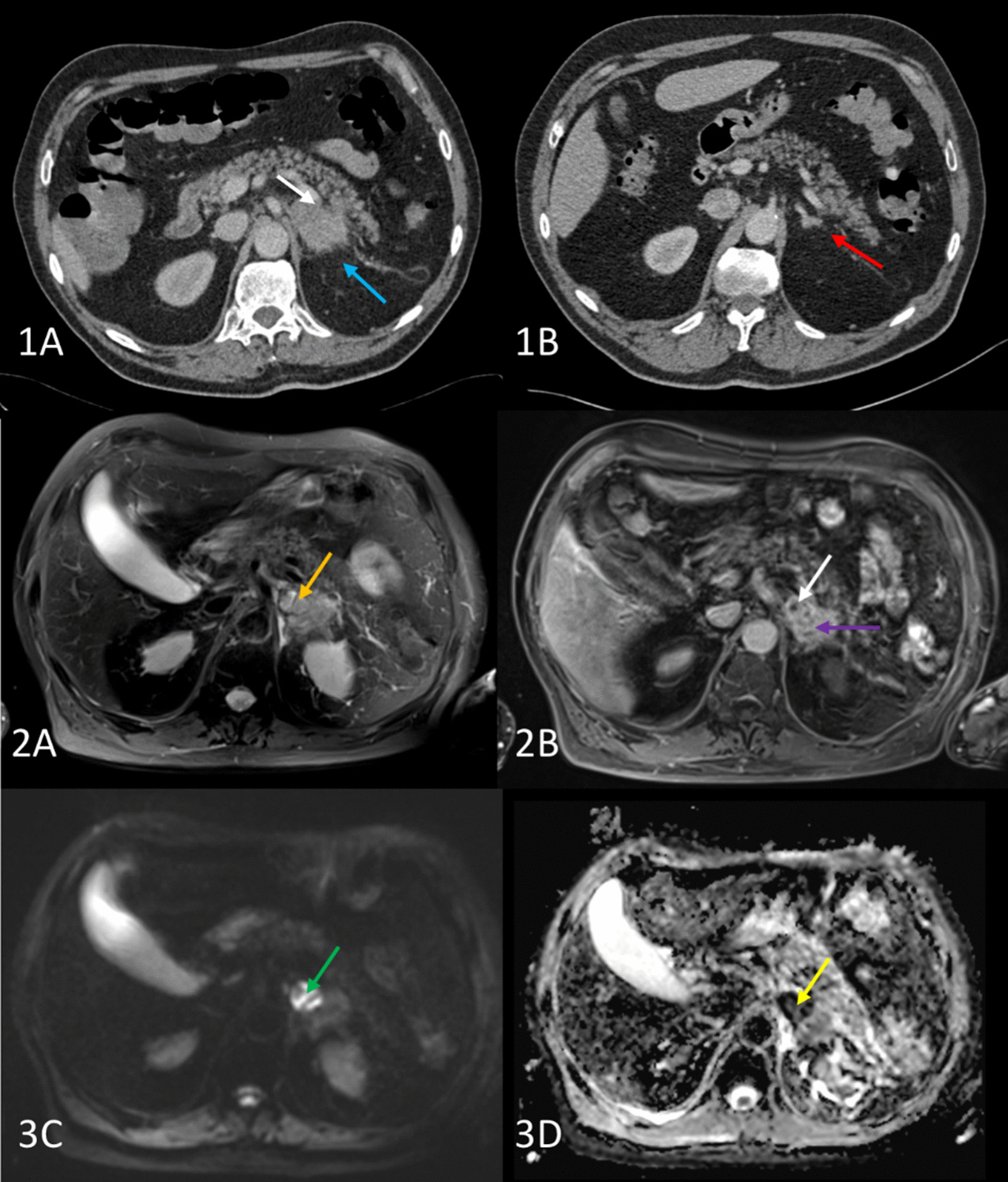
Abdominal CT and MR scan. Abdominal CT scan [1] in axial view, initial (1A) and 6 months after starting antibiotic treatment (1B). The large left adrenal abscess (white arrow) is accompanied by inflammation of the surrounding fat (blue arrow). Note the normalization of the left adrenal gland (red arrow) and the disappearance of the inflammation of the surrounding fat. MR images [2] acquired during the antibiotic treatment. The T2 with fat saturation images (2A) shows a hypersignal in the anterior part of the left adrenal gland (orange arrow). On the enhanced T1 sequence (2B), this image is not enhanced (white arrow) corresponding to the central part of the abscess (necrosis), while the rest of the adrenal gland is normally enhanced (purple arrow). The hypersignal in diffusion sequence (3C, green arrow), especially with a very low ADC (apparent diffusion coefficient) (3D), here less than 0.5·10^− 6^mm^2^ s^− 1^ (yellow arrow), leading to the diagnosis of abscess

A cerebral CT-scan was performed and revealed bilateral lesions with circumferential contrast enhancement and edema. The biggest lesion (13 mm) was in the right occipital lobe. A chest, abdomen and pelvis (C.A.P) CT-scan showed a left adrenal poly-lobed nodular hypertrophy of 57 × 33 × 62 mm (Fig. 1). A Positron Emission Tomography scanner was also made, showing hypermetabolic left adrenal lesion (standardized uptake value (SUV) max = 14), pulmonary hypermetabolism in bilateral postero-basal regions and pleural effusions (SUV max = 4.3), and no other suspect lesion. An abdominal magnetic resonance imaging (MRI) was performed to help distinguishing the nature of this adrenal hypertrophy (Fig. 1). The lesion was clearly in favor of a left adrenal abscess because of the MRI characteristic and the size regression of the lesion under antimicrobial treatment. Transthoracic and trans-esophageal echocardiography showed no sign of endocarditis. HIV serology was negative but a CD4+ T lymphopenia (402 cell/µL or 38%) was observed. Serum protein electrophoresis revealed a normal rate of gamma globulin (10 g/L) with no monoclonal spike. The patient was not diabetic.

Gradually, the patient’s clinical condition improved and two weeks after admission, he was transferred to the infectious disease department. Antibiotic treatment was changed to TMP-SMX (4.8 g/960 mg/24 h) and cefotaxime (12 g/24 h for one week and then 9 g/24 h). Nineteen days after beginning these antibiotics, the patient developed skin rash, justifying stopping TMP-SMX and replacing it by clarithromycin (1.5 g/24 h). Six weeks after the beginning of the antibiotic treatment (December 16th, 2017), the patient has recovered, cefotaxime was replaced by ceftriaxone (4 g/24 h) and he returned home. After three months of antibiotic treatment (February 5th, 2018), ceftriaxone was changed to doxycycline (200 mg/24 h) but still combined with clarithromycin (1.5 g/24 h). A C.A.P and brain CT scan were performed after six months of treatment (April 23th, 2018) and showed a complete regression of all lesions including the left adrenal gland with restitution *ad integrum* and antibiotics were discontinued (Fig. 1).

## Discussion and conclusion

We report here a rare case of *Nocardia cyriacigeorgica* disseminated infection in a non-immunocompromised man with notably a bacteremia and an adrenal gland abscess. The species involved in this case was not a new species as firstly suspected by Yassin et al. [7], nor an emerging pathogen, but in fact, *N. cyriacigeorgica* and *N. asteroides* drug pattern type VI belong to the same species [8]. It is no longer appropriate to mention the *Nocardia asteroides* complex and its six «drug pattern type» whom now are six different taxa: *N. abscessus* (drug pattern I), *N. brevicatena /N. paucivorans* (drug pattern II), *N. nova* complex (drug pattern III), *N. transvalensis* complex (drug pattern IV), *N. farcinica* (drug pattern V) and *N. cyriacigeorgica* (drug pattern VI) [3]. Thus, the main *Nocardia* species isolated in the majority of human infections are part of the former *N. asteroides* complex : *N. farcinica*, *N. abscessus*, *N. cyriacigeorgica*, and *N. nova* [4, 9, 10] including cases reported prior to the availability of molecular tests [3, 8]. In our case, *N. cyriacigeorgica* was identified by MALDI-TOF MS. Although progress is still needed for the identification of uncommon species, MALDI-TOF MS is a valuable aid for more accurate and rapid identification of *Nocardia* species [3]. This easier identification probably helps physicians to be more familiar to the complexity of *Nocardia* taxonomy [3].

Even though *Nocardia* species can grow in a variety of blood culture media, bacteremia during Nocardiosis is rarely reported [5]. We reviewed large series of *Nocardia* infection published [10–17] and found that bacteremia frequency range from 3.7 to 26.5% with a mean value of 10,1%. The study reporting the higher rate of bacteremia (26.5%) included mainly immunocompromised patients with 50% of disseminated forms [10]. Blood cultures seem then useful in the diagnosis of Nocardiosis. In the review of Kontoyiannis et al. [5], the isolation of *Nocardia* in blood culture preceded the isolation from other sites in 44% (8/18) of cases and blood cultures were the only source of diagnostic material in one-fourth of the cases (9/36). Thus, detection of *N. cyriacigeorgica* in blood cultures allowed a quick diagnosis and accurate treatment that probably improved the prognosis of our patient.

Low bacteremia frequency during *Nocardia* could be explained by a too short incubation time of blood cultures, a co-infection with more resilient organisms (as Gram-negative bacteria), an infrequent and intermittent bacteremia or an empiric treatment with good activity against *Nocardia* [5, 18]. Nowadays, usual blood culture media allows the detection of *Nocardia* spp., although specific media increase detection of *Nocardia* spp. such as biphasic brain-heart infusion or Castañeda media. In our case, the bacteremia was detected using BD BACTEC FX (Becton Dickinson®). The growth of bacteria on usual blood culture probably increases the diagnosis of bacteremia in this infection. To increase awareness about *Nocardia* infections in microbiology laboratories and to improve the yield of cultures for *Nocardia,* it may be advisable to increase duration of incubation and perform subcultures on to blood agar from the broth [5, 18].

Hematogenous dissemination of Nocardiosis can affect all organs, but some are rarely involved such as the adrenal glands. Only fourteen cases of *Nocardia* adrenal abscesses are reported in the literature. Adrenal abscesses are caused by *N. farcinica* (7/14), *N. asteroides* (4/14) and *N. beijingensis* (1/14) (see Table 1) [6, 10, 11, 19–22]. *N. cyriacigeorgica* is involved in only two cases [19, 21]. Adrenal masses are benign or malignant tumours most of the time but infectious causes (abscesses) must also be considered [6]. Disseminated Nocardiosis with adrenal abscesses can masquerading as malignant disease and commonly leading to an invasive strategy (puncture, laparoscopic drainage, adrenalectomy) to make a diagnosis and can lead to injury (see Table 1) [6, 10, 11, 19–22]. In our case, the presence of bacteremia, the use of medical imaging and biology and the evolution under treatment (lesion with progressive regression and *restitution ad integrum* after 6 months of antibiotic regimen) helped us to confirm infectious nature of the adrenal mass. Among the cases of *Nocardia* adrenal abscess [6, 10, 11, 19–22], seven were associated with bacteremia (7/14 i.e. 50%) [6, 10, 11, 19, 20, 23, 24] and only one did not underwent an invasive procedure for diagnosis [23]. Furthermore, in a recent similar case, the patient underwent adrenalectomy and blood cultures taken 3 days prior to surgery grew *N. cyriacigeorgica* [21]. When we face an adrenal tumour with associated lesions (lung, brain etc.), it is therefore important to take time and promote non-invasive strategies such as medical imaging and biology (blood cultures particularly) to make a diagnosis. Moreover, even if some authors suggested that draining abscess collection is crucial to a successful outcome [5], in our case, a strategy with antibiotic regimen only was successful.

**Table 1:** Cases of *Nocardia* spp. adrenal abscesses reported in literature (NR: not reported)

References	Years	Patients characteristics	Symptoms	Disseminated Nocardiosis	Adrenal abscess side/Nocardia species	Treatment	Outcome
Kim et al. [25]	1991	Male, 38 years, AIDS	Left upper quadrant abdominal pain, fever, chills	Paraaortic and mesenteric lymphadenopathy	Left/*N. asteroides*	Cefoxitin Surgical drainage of the mass	Died
Arabi et al. [23]	1996	Male, 39 years, AIDS	Epigastric pain, fever, vomiting	Brain, bacteremia	Bilateral/*N. asteroides*	Cefotetan, gentamicin, doxycycline then TMP-SMX, imipenem, amikacin, and ciprofloxacin	Survived
Midiri et al. [26]	1998	Female, 49 years, rheumatoid arthritis treated by corticosteroid therapy	Fever and left flank plain	Lung, spleen	Left/*N. asteroides*	TMP-SMX Left adrenalectomy	Survived
Moiton et al. [11]	2002	Male, 69 years, active cancer, steroids	Pneumoniae, aphasia, motor deficit	Lung, brain, bacteremia	NR/*N. farcinica*	NR	Died
Chong et al. [27]	2004	Male, 34 years, AIDS	Fever, left loin pain, hematuria	No other location	Left/*N. asteroides*	Ceftriaxone, TMP-SMX Laparoscopic drainage	Survived
Elsayed et al. [19]	2005	Female, 69 years, type 2 diabetes mellitus, chronic lymphocytic leukemia, hypogammaglobulinemia	Malaise, right-sided flank and pleuritic chest discomfort, left leg weakness, ataxic gait	Lung, brain, bacteremia	Right/*N. cyriacigeorgica*	Meropenem, TMP-SMX	Survived (mild to moderate speech impairment, generalized weakness)
Haussaire et al. [10]	2008	Male, 35 years, kidney transplant, immunosuppressor treatment (calcineurin inhibitor, antimetabolite, steroids)	NR	Lung, kidney, pancreas, adrenal glands, bacteremia	NR/*N. farcinica*	TMP-SMX, carbapenem, amikacin	Survived (hypoacusis)
Tachezy et al. [28]	2009	Female, 71 years, former alcohol addiction, malnutrition	Fever, non-productive cough, adynamia	Lung, inferior vena cava, right hepatic and renal capsule, diaphragm, brain	Right/*N. farcinica*	Imipenem, amikacin, TMP-SMX then TMP-SMX alone Right adrenalectomy with resection of IVC diaphragm, retroperitoneum and Gerota’s fascia	Survived
Al-Tawfiq and Al-Khatti [29]	2010	Female, 66 years, psoriasis treated by TNF-alpha therapy, type 2 diabetes	Fever, chills, profuse sweating, left upper quadrant abdominal pain	Left renal vein, spleen, retroperitoneal and mesenteric lymphadenopathies	Bilateral/*N. farcinica*	Vancomycin, meropenem then TMP-SMX, linezolid Aspiration and biopsy of the left adrenal lesion	Died
de Montmollin et al. [24]	2012	Female, 59 years, chronic parenteral nutrition, malnutrition, hemodialysis	Fever, right lumbar region pain	Bacteremia	Right/*N. farcinica*	Amikacin, cefuroxime then TMP-SMX Echography-guided aspiration and drainage	Died
Jackson et al. [6]	2017	Male, 69 years	Fever, night sweats, left upper quadrant abdominal pain	Lung, central nervous system, bone marrow, left renal vein, bacteremia, urine	Left/*N. farcinica*	Meropenem, TMP-SMX	Survived
Jackson and Shorman [20]	2018	Male, 39 years, splenectomy, intravenous drug user	Fever, generalized weakness, abdominal pain, orthostatic symptoms	Heart, bacteremia	Bilateral/*N. farcinica*	Meropenem, TMP-SMX Bilateral percutaneous drains in the adrenal glands	Survived (at 4 weeks of treatment, then lost to follow-up)
Langmaid et al. [21]	2020	Male, 35 years, immunocompetent	Fever, dry cough and left-sided abdominal pain	Lung	Left/*N. cyriacigeorgica*	Meropenem, TMP-SMX then ceftriaxone and TMP-SMX Left adrenalectomy	Survived
Pender et al. [22]	2022	Male, 57 years, poorly controlled type 2 diabetes mellitus	Left upper quadrant abdominal pain, subjective fevers, diaphoresis, tachycardia, anorexia, nausea, weight loss	Lung	Left/N. *beijingensis*	TMP-SMX, linezolid Left adrenalectomy	Survived
This case	2017	Male, 77 years, no predisposing factors	Deterioration of general status, depressive syndrome, respiratory distress	Lung, brain, bacteremia	Left/*N. cyriacigeorgica*	Cefotaxime, TMP-SMX then cefotaxime, clarithromycin then doxycycline and clarithromycin	Survived

In conclusion, the *Nocardia* spp. taxonomy underwent many changes in recent years and despite complexity, knowledge of most common species is needed. *Nocardia* bacteremia is a rare event but blood cultures may help to improve detection of *Nocardia* spp. in a non-invasive way. Despite *Nocardia* spp. adrenal abscess being very rare with only fourteen cases reported, it is a true cause of adrenal masses. Our report suggests that clinician should be aware of this rare location and prioritize a non-invasive strategy.

## Data Availability

Not applicable.

## Abbreviations

Hsp65: Heat shock protein
rRNA: Ribosomal ribonucleic acid
CT: Computerized tomography
MALDI-TOF MS: Matrix-assisted laser desorption-ionization-time of flight mass spectrometry
TMP-SMX: Trimethoprim-sulfamethoxazole
C.A.P: Chest, abdomen and pelvis
SUV: Standardized uptake value
MRI: Magnetic resonance imaging
HIV: Human immunodeficiency virus

## References

[1] Brown-Elliott BA, Brown JM, Conville PS, Wallace RJ (2006). Clinical and laboratory features of the Nocardia spp. based on current molecular taxonomy. Clin Microbiol Rev.

[2] Saubolle MA, Sussland D, Nocardiosis (2003). J Clin Microbiol.

[3] Conville PS, Brown-Elliott BA, Smith T, Zelazny AM (2018). The complexities of Nocardia taxonomy and identification. J Clin Microbiol.

[4] Rodriguez-Nava V, Zoropoguy A, Laurent F, Blaha D, Couble A, Mouniée D (2008). La nocardiose, une maladie en expansion. Antibiotiques..

[5] Kontoyiannis DP, Ruoff K, Hooper DC (1998). Nocardia bacteremia. Report of 4 cases and review of the literature. Med (Baltim).

[6] Jackson C, McCullar B, Joglekar K, Seth A, Pokharna H (2017). Disseminated *Nocardia farcinica* pneumonia with left adrenal gland abscess. Cureus.

[7] Yassin AF, Rainey FA, Steiner U (2001). *Nocardia cyriacigeorgici* sp. nov. Int J Syst Evol Microbiol.

[8] Conville PS, Witebsky FG (2007). Organisms designated as *Nocardia asteroides* drug pattern type VI are members of the species *Nocardia cyriacigeorgica*. J Clin Microbiol.

[9] Wang HL, Seo YH, LaSala PR, Tarrand JJ, Han XY (2014). Nocardiosis in 132 patients with cancer: microbiological and clinical analyses. Am J Clin Pathol.

[10] Haussaire D, Fournier PE, Djiguiba K, Moal V, Legris T, Purgus R (2017). Nocardiosis in the south of France over a 10-years period, 2004–2014. Int J Infect Dis IJID Off Publ Int Soc Infect Dis.

[11] Moiton MP, Robert D, Bébéar CM, Neau D, Dugué C, Ragnaud JM (2006). Clinical, microbiological, and therapeutic aspects of Nocardia sp. infections in the Bordeaux hospital from 1993 to 2003. Med Mal Infect.

[12] Majeed A, Beatty N, Iftikhar A, Mushtaq A, Fisher J, Gaynor P (2018). A 20-year experience with nocardiosis in solid organ transplant (SOT) recipients in the Southwestern United States: a single-center study. Transpl Infect Dis Off J Transplant Soc.

[13] Chen J, Zhou H, Xu P, Zhang P, Ma S, Zhou J (2014). Clinical and radiographic characteristics of pulmonary nocardiosis: clues to earlier diagnosis. PLoS ONE.

[14] Yang M, Xu M, Wei W, Gao H, Zhang X, Zhao H (2014). Clinical findings of 40 patients with nocardiosis: a retrospective analysis in a tertiary hospital. Exp Ther Med.

[15] Peleg AY, Husain S, Qureshi ZA, Silveira FP, Sarumi M, Shutt KA (2007). Risk factors, clinical characteristics, and outcome of Nocardia infection in organ transplant recipients: a matched case-control study. Clin Infect Dis Off Publ Infect Dis Soc Am.

[16] Castro JG, Espinoza L (2007). Nocardia species infections in a large county hospital in Miami: 6 years experience. J Infect.

[17] Mootsikapun P, Intarapoka B, Liawnoraset W (2005). Nocardiosis in Srinagarind Hospital, Thailand: review of 70 cases from 1996–2001. Int J Infect Dis IJID Off Publ Int Soc Infect Dis.

[18] Lederman ER, Crum NF (2004). A case series and focused review of nocardiosis: clinical and microbiologic aspects. Medicine..

[19] Elsayed S, Kealey A, Coffin CS, Read R, Megran D, Zhang K (2006). Nocardia cyriacigeorgica septicemia. J Clin Microbiol.

[20] Jackson LE, Shorman M (2018). A case of bilateral *Nocardia francinia* adrenal abscesses in an intravenous drug-using splenectomized patient with tricuspid endocarditis. Open Forum Infect Dis.

[21] Langmaid T, Jassal K, Meher-Homji Z, Lee JC, Serpell J, Yeung M (2021). Disseminated nocardiosis with adrenal abscess masquerading as metastatic adrenal cancer in an immunocompetent adult. ANZ J Surg.

[22] Pender M, Mehta N, Hamilton BD, Swaminathan S (2022). *Nocardia beijingensis* isolated from an adrenal abscess in a diabetic host. Open Forum Infect Dis.

[23] Arabi Y, Fairfax MR, Szuba MJ, Crane L, Schuman P (1996). Adrenal insufficiency, recurrent bacteremia, and disseminated abscesses caused by *Nocardia asteroides* in a patient with acquired immunodeficiency syndrome. Diagn Microbiol Infect Dis.

[24] de Montmollin E, Corcos O, Noussair L, Leflon-Guibout V, Belmatoug N, Joly F (2012). Retroperitoneal abscesses due to *Nocardia farcinica*: report of two cases in patients with malnutrition. Infection.

[25] Kim J, Minamoto GY, Grieco MH (1991). Nocardial infection as a complication of AIDS: report of six cases and review. Rev Infect Dis.

[26] Midiri M, Finazzo M, Bartolotta TV, Maria MD (1998). Nocardial adrenal abscess: CT and MR findings. Eur Radiol.

[27] Chong YL, Green JA, Toh KL, Tan JK (2004). Laparoscopic drainage of nocardial adrenal abscess in an HIV positive patient. Int J Urol Off J Jpn Urol Assoc.

[28] Tachezy M, Simon P, Ilchmann C, Vashist YK, Izbicki JR, Gawad KA (2009). Abscess of adrenal gland caused by disseminated subacute *Nocardia farcinica* pneumonia. A case report and mini-review of the literature. BMC Infect Dis..

[29] Al-Tawfiq JA, Al-Khatti AA (2010). Disseminated systemic *Nocardia farcinica* infection complicating alefacept and infliximab therapy in a patient with severe psoriasis. Int J Infect Dis IJID Off Publ Int Soc Infect Dis.

